# Ecosystem responses to climate change at a Low Arctic and a High Arctic long-term research site

**DOI:** 10.1007/s13280-016-0870-x

**Published:** 2017-01-23

**Authors:** John E. Hobbie, Gaius R. Shaver, Edward B. Rastetter, Jessica E. Cherry, Scott J. Goetz, Kevin C. Guay, William A. Gould, George W. Kling

**Affiliations:** 1 Ecosystems Center, Marine Biological Laboratory, Woods Hole, MA 02543 USA; 2 International Arctic Research Center, University of Alaska, Fairbanks, AK 99775 USA; 3 Woods Hole Research Center, Falmouth, MA 02540 USA; 4 International Institute of Tropical Forestry, Río Piedras, PR 00926 USA; 5 Department of Ecology and Evolutionary Biology, University of Michigan, Ann Arbor, MI 48109 USA

**Keywords:** Alaska Toolik, Climate change, Ecological effects, Greenland Zackenberg, Medium pass filter, Vegetation

## Abstract

Long-term measurements of ecological effects of warming are often not statistically significant because of annual variability or signal noise. These are reduced in indicators that filter or reduce the noise around the signal and allow effects of climate warming to emerge. In this way, certain indicators act as medium pass filters integrating the signal over years-to-decades. In the Alaskan Arctic, the 25-year record of warming of air temperature revealed no significant trend, yet environmental and ecological changes prove that warming is affecting the ecosystem. The useful indicators are deep permafrost temperatures, vegetation and shrub biomass, satellite measures of canopy reflectance (NDVI), and chemical measures of soil weathering. In contrast, the 18-year record in the Greenland Arctic revealed an extremely high summer air-warming of 1.3 °C/decade; the cover of some plant species increased while the cover of others decreased. Useful indicators of change are NDVI and the active layer thickness.

## Introduction

Climate warming in the Arctic, substantial over recent decades and well-documented in IPCC reports (IPCC 2001, 2013), is reflected in changes in a wide range of environmental and ecological measures. These illustrate convincingly that the Arctic is undergoing a system-wide response (ACIA 2005; Hinzman et al. 2005). The changing measures range from physical state variables, such as air temperature, permafrost temperature (Romanovsky et al. 2010), or the depth of seasonal thaw (Goulden et al. 1998), to changes in ecological processes, such as plant growth, which can result in changes in the state of ecosystem components such as plant biomass or changes in ecosystem structure (Chapin et al. 2000; Sturm et al. 2001; Epstein et al. 2004). In spite of the large number of environmental and ecological measurements made over recent decades, it has proven difficult to discover statistically significant trends in these measurements. This difficulty is caused by the high annual and seasonal variability of warming in the air temperature and the complexity of biological interactions.

**Fig. 1:**
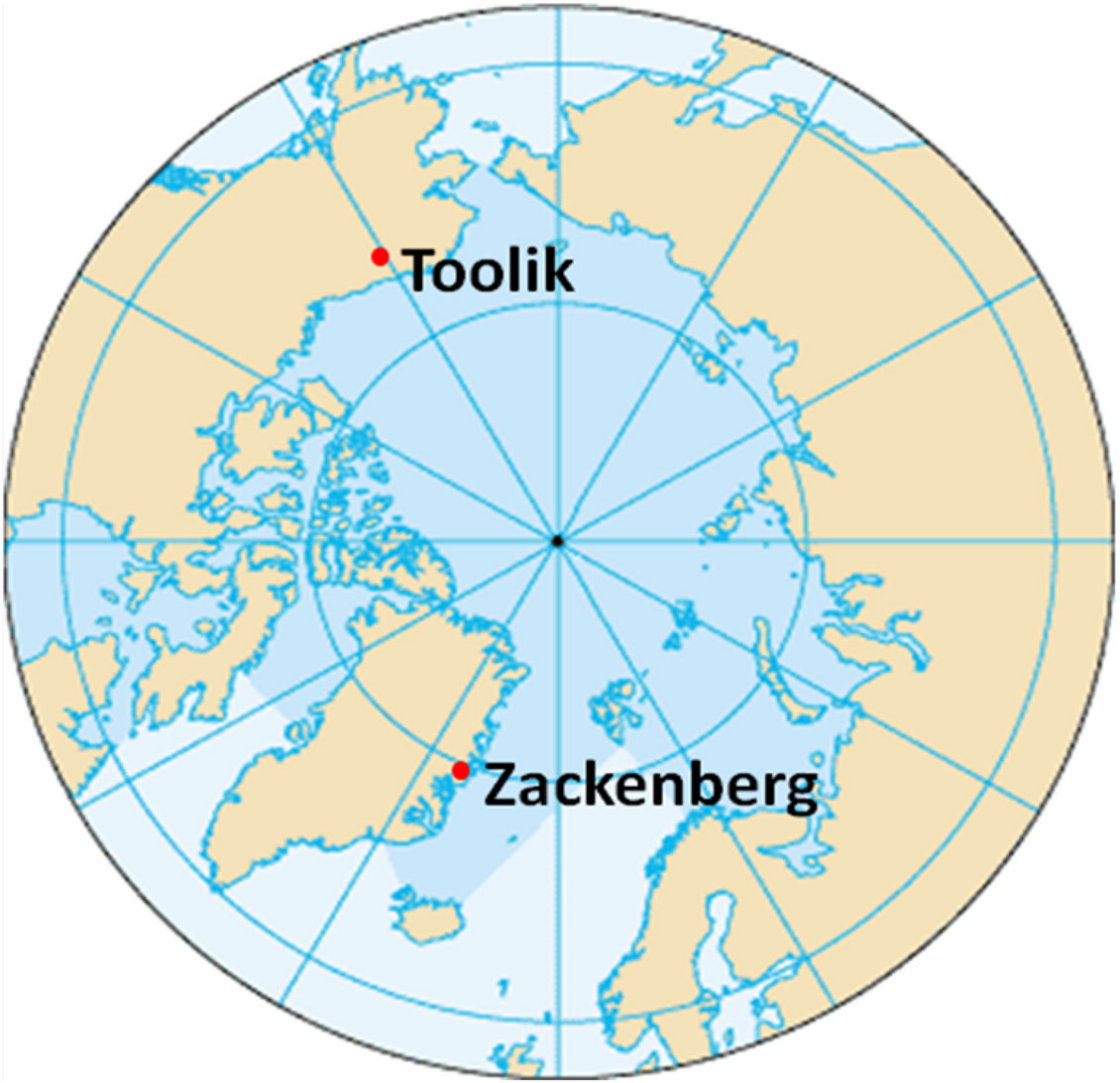
Location of Toolik, Alaska (68^o^38′N, 149^o^43′W) and Zackenberg, Greenland (74^o^30′N, 21^o^30′W), long-term arctic study sites

One solution to the variability problem is to carry out long-term studies. These studies are expensive to carry out in the Arctic with the result that many detailed studies have been relatively short-term (e.g., the IBP Arctic projects in the U.S. and Canada), or have been long-term projects limited in scope (e.g., the Sub-Arctic Stordalen project in Abisko, Sweden; Jonasson et al. 2012). Currently, there are but two projects underway that are both long-term and broad in scope: Toolik in the Low Arctic of northern Alaska and Zackenberg in the High Arctic of northeast Greenland (Fig. 1). Here we use data from these sites to ask which types of measures actually yield statistically significant trends of effects of climate warming? Further, are there common characteristics of these useful measures that reduce variability?

## Study sites

The Toolik project (Table 1) is located at the University of Alaska’s Toolik Field Station (TFS) some 125 km inland from the Arctic Ocean. The Long Term Ecological Research (LTER)^1^ and related projects at this site have collected data on a wide variety of variables since 1975 (Hobbie 2014). The long-term research site in the Zackenberg Valley (Table 1) is located on the coast of northeast Greenland where environmental and ecological data have been collected since 1995 (National Environmental Research Institute, Aarhus University^2^).

**Table 1:** Ecological settings for Toolik and Zackenberg research sites

	Toolik field station	Zackenberg
Location	Inland, Northern Alaska 68^o^38′N, 149^o^43′W, 719 m altitude	Coast, Northeast Greenland 74^o^30′N, 21^o^30′W, 0 m altitude
Physical setting	Rolling foothills, Continuous permafrost (200 m), annual temperature −8 °C, summer (mid-June to mid-August) 9 °C, annual precipitation 312 mm	Mountain valley, Continuous permafrost (estimated 200–400 m), annual temperature −8 °C, summer (3 months) 4.5 °C, annual precipitation 261 mm
Ecology	Tussock tundra (sedges, evergreen and deciduous shrubs, forbs, mosses, and lichens). Low shrubs, birches, and willows grow between tussocks and along water tracks and stream banks. Low Arctic	Central valley floor dominated by Ericaceous evergreen (*Cassiope tetragona*), by heaths and arctic willow (*Salix arctica*)j, and by snow-beds, grasslands, and fens. This High Arctic ecosystem has relatively low biodiversity and low species redundancy
Projects	LTER (Long Term Ecological Research), ITEX (International Tundra Experiment), NOAA’s Arctic Program, CALM (Circumpolar Active Layer Monitoring), and the TFS environmental monitoring program	BioBasis programme of NERI, Danish Environmental Protection Agency, CALM (Circumpolar Active Layer Monitoring), ECOGLOBE (Aarhus University), INTERACT, World Wildlife Fund, GeoBasis, NARP

Both sites are underlain by hundreds of meters of continuous permafrost and have similar average annual temperatures of ~ −8 °C. Summers, however, are shorter and cooler at Zackenberg (4.5 °C) than at Toolik (9 °C). The short and cool summers of the Zackenberg valley restrict the number of vascular plant species in the dominant moist heath tundra so this High Arctic site has a relatively low biodiversity (Callaghan 2005; Schmidt et al. 2012). In contrast, the rolling uplands at the Low Arctic Toolik site are dominated by dwarf-shrub heath-tussock tundra and have many more plant species. Bliss (1997) surveyed the North American Arctic, including Greenland, and reported that the High Arctic has 300 species, mostly herbaceous forms, while the Low Arctic has 700 species, including a number of woody species such as birch and willow.

## Materials and methods

### Environmental and ecological monitoring at Toolik and Zackenberg

The monitoring program at Toolik includes measurements on streams, lakes, and tundra (Table 2). In this article, we include results of permafrost temperatures, vegetation growth, thaw depth, and lake alkalinity (Cherry et al. 2014; Shaver et al. 2014; Kling et al. 2014), extend the air temperature data, and add long-term satellite measures of plant biomass. The monitoring program of tundra and lakes at Zackenberg includes climate, the thickness of the active layer, plant community abundance, and productivity, and trends in terrestrial and freshwater ecosystem components.

**Table 2:** Environmental and ecological variables measured over the long-term at Toolik and Zackenberg sites

Site	Environmental and ecological variables
Toolik Site	
Climate	Air temperature, precipitation, wind speed and direction, and growing season dates for 1989–2010 are in Cherry et al. (2014)
Thaw depth	Summer depths of thaw for July and August in the Tussock Watershed, 1989–2010, are in Kling et al. (2014)
Biology	Net primary production aboveground for moist acidic tundra from 6 harvests 1989–2000 and point-frame data (4 harvests 1989–2008) are in Shaver et al. (2014)
Kuparuk River	
Climate, physics	Climatic norms for river basin (1989–2010) and discharge and temperature (1972–2010) are in Bowden et al. (2014)
Biology	Primary production and respiration (1984–1998), epilithic chlorophyll (1983–2010), bryophyte cover (1992–2006), benthic insect taxa (1984–1998), and grayling growth (1985–2005) data are in Bowden et al. (2014)
Toolik Lake	
Physics and chemistry	Epilimnion temperature (July, 1985–2007) and summer alkalinity (1975–2011) data are in Luecke et al. (2014)
Biology	Chlorophyll (July, 1985–2010) data are in Luecke et al. (2014)
Zackenberg	
Physics	Temperature, 1991–2005, wind direction and speed (1985–2005), and precipitation, 1997–2005, are given in Hansen et al. (2008). Data are available at Greenland Ecosystem Monitoring (http://www.data.g-e-m.dk)
Thaw depth	The summer thaw depth progression from June 1 to September 7 at ZEROCALM-2, 1996–2005, is given in Christiansen et al. (2008)
Plant communities and production	Plant communities were analyzed (1997, 2008) in relation to summer temperature and spring snow cover. Five replicate plots in eight plant communities were sampled (Schmidt et al. 2012). NDVI measures (Tagesson et al. 2012) gave gross primary production at the peak of the growing season from 1992 to 2008
Variations and trends in biotic and abiotic ecosystem compartments	Precipitation, temperature, and snow depth measured hourly (1996–2010). Abundance of 6 plant species, 6 taxa of arthropods, 4 species of birds, and 3 mammals measured weekly and seasonally (Mortensen et al. 2014). At 2 lakes, temperature, ice cover, and nutrients were measured (1997–2005) as well as volume of phytoplankton and abundance of zooplankton (Christoffersen et al. 2008)

### Methods for data from Toolik

**Fig. 2:**
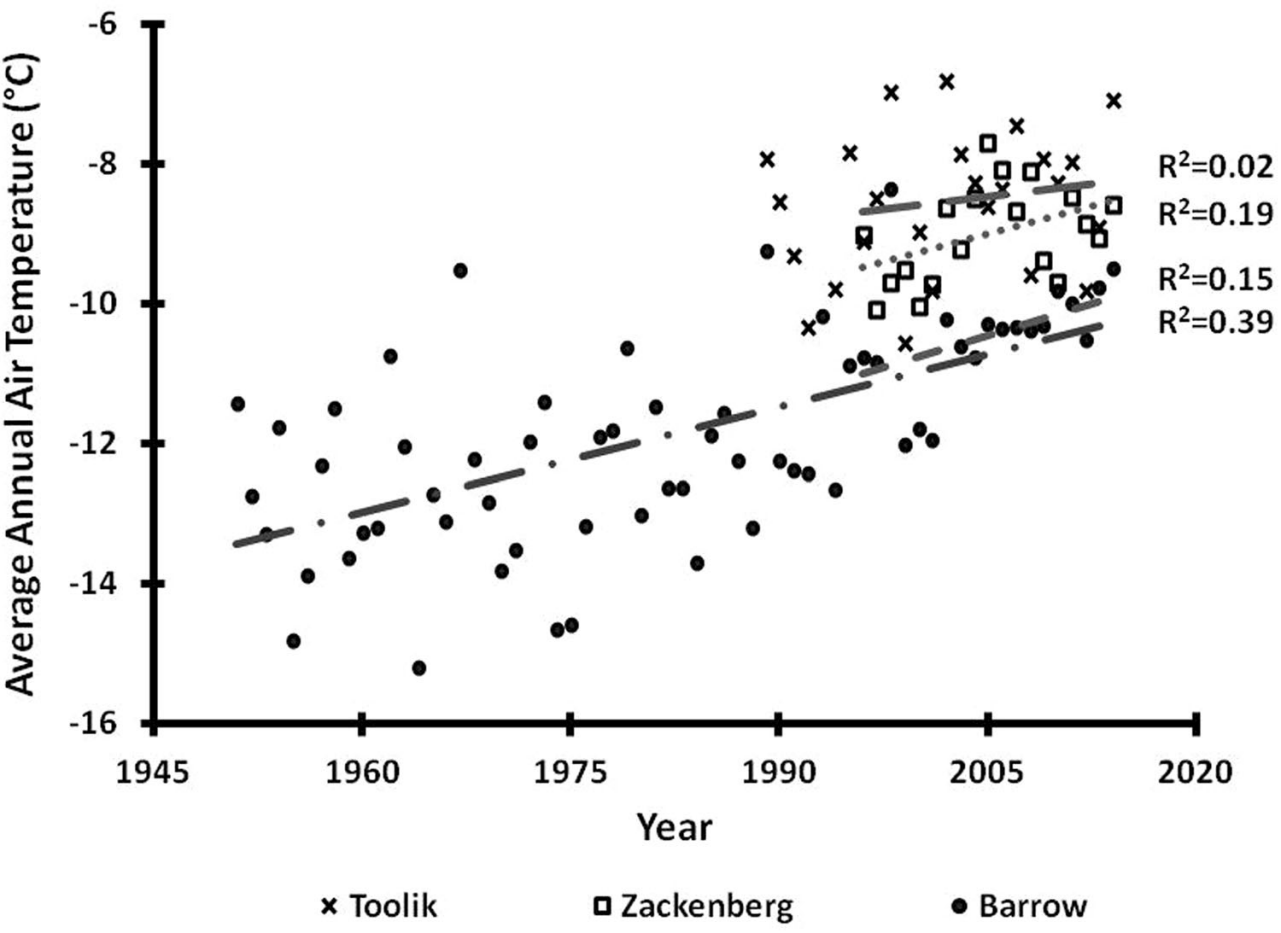
Annual mean Barrow SAT (surface air temperature, *closed circles*) for 1950–2014, annual mean Toolik SAT (x’s) for 1989-2014, and Zackenberg SAT (*open squares*) for 1996–2014. Also shown are the linear regressions for Barrow 1950–2014 (*dashed dot line*), Barrow 1996–2014 (*short-dashed line*), Toolik 1996–2014 (*long-dashed line*), and Zackenberg 1996–2014 (*dotted line*). Regression lines and coefficients are ordered from top to bottom as Toolik, Zackenberg, Barrow (1996–2014), and Barrow (1950–2014). Only the Barrow 1950–2014 and Zackenberg 1996–2014 linear regressions are significant (p < 0.01). Data from Alaska Climate Research Center (2015), Toolik LTER (http://dx.doi.org/10.6073/pasta/2f655c865f42136611b2605ae778d275), and Zackenberg (http://www.data.g-e-m.dk)

**Fig. 3:**
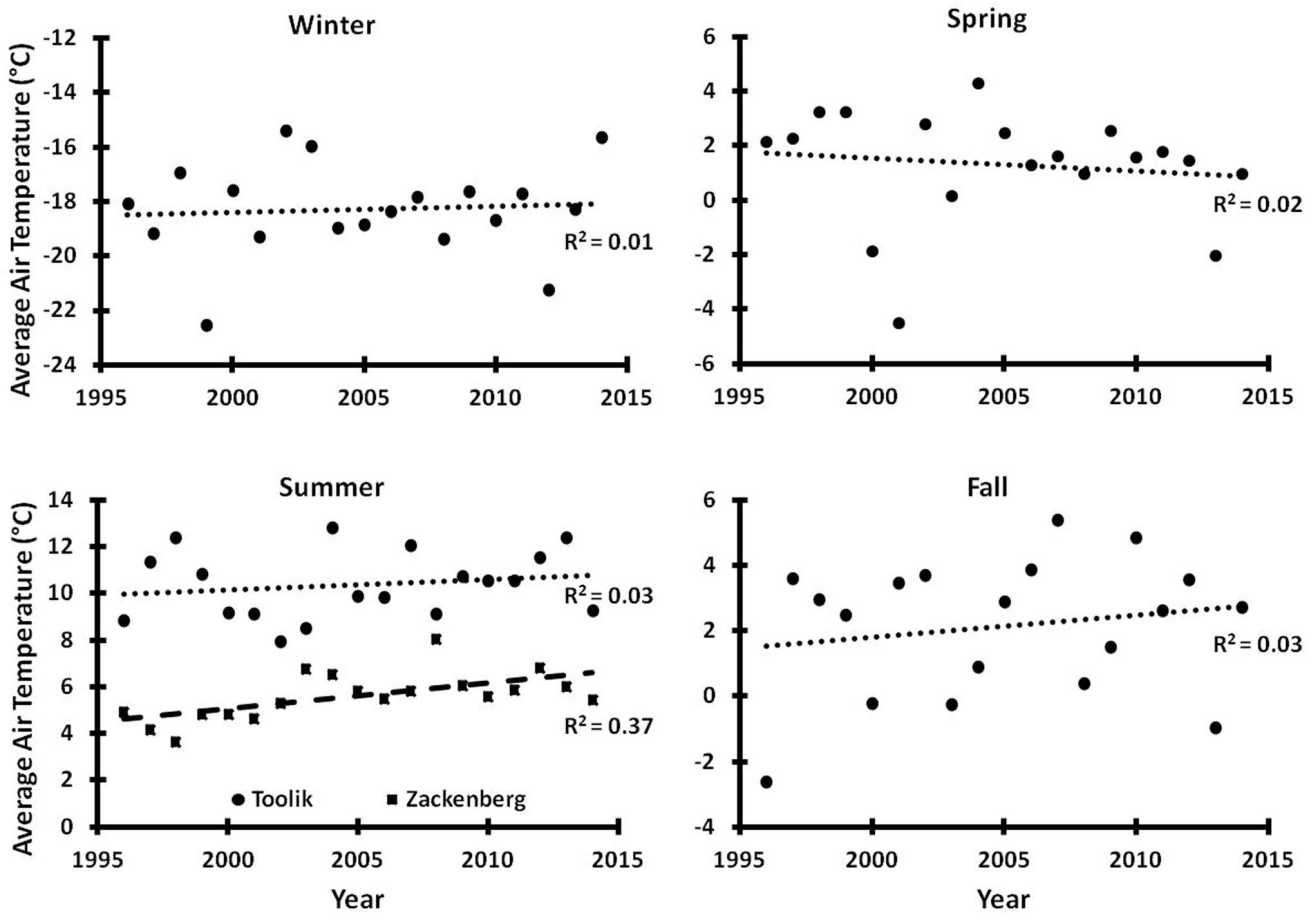
Seasonal means of Toolik LTER SAT 1988–2014 for winter (October 1–April 30), spring (May 1–June 15), summer (June 16–August 15), and fall (August 16–September 30). Summer data also include 1996–2014 means from Zackenberg (*closed squares*) from August 16 to September 30. Trend lines are linear regressions; only Zackenberg summer trends are significant (p < 0.01). Data sources same as in Fig. 2

Cherry et al. (2014) described the surface air temperature (SAT) for the Toolik Field Station for the period 1989–2010 (Fig. 2). Here we update the annual data through 2014 (Fig. 2) and also separately analyze the air temperature in winter, spring, summer, and fall seasons (Fig. 3). Romanovsky et al. (2010) measured permafrost temperatures once a year since 1983 at a depth of 20 m in boreholes along the Dalton Highway.

As a part of the international CALM program (Circumpolar Active Layer Monitoring described in Brown et al. 2000), summer thaw depth of the active layer in moist acidic tundra at Toolik was measured using steel probes at 96 individual sites within a 200 × 900 m grid. At each site, three measurements were averaged, and a grand average of all sites was calculated for each of two dates in summers from 1990 to 2011. Additional information on thawing the soil came for measures of alkalinity in Toolik Lake. Alkalinity was determined by potentiometric titration (Kling et al. 1992, 2000) and was averaged across depth and season to provide an annual estimate. Keller et al. (2010) measured strontium isotope ratios (^87^Sr/^86^Sr), which decrease with depth in soils at the Arctic LTER, to estimate the increasing depth of water flow within the soil.

Using the point-frame method described by Walker (1996), Gould and Mercado-Díaz (in Shaver et al. 2014) monitored the response of plant communities to ambient climate in 155 permanent plots. Measurements were made at 5- to 7-year intervals since 1989 in two 1 km^2^ grids set up by Walker et al. (1989) at Toolik Lake and nearby Imnavait Creek. This monitoring was a part of the International Tundra Experiment (ITEX).

Guay et al. (2014) analyzed satellite data to determine annual dynamics of normalized-difference vegetation index (NDVI), a measure of plant productivity, which is also highly correlated with aboveground biomass in arctic systems (Boelman et al. 2003; Raynolds et al. 2012). The NDVI data were derived from the GIMMS-AVHRR times series, version 3 g (Pinzón and Tucker 2014), with a 0.07^o^ (8 km) spatial resolution. We analyzed the GIMMS-3 g dataset across the years 1982–2014 for a 40-km (20 km radius) area surrounding the Toolik Field Station. Seasonal periods of NDVI trends through time were consistent with the seasonal periods used to assess trends in air temperature (see legend for Fig. 3).

## Results

### Climate trends: Arctic, North Slope of Alaska, Toolik, and Zackenberg

Over the entire Arctic, the average SAT for the past century increased by approximately 0.09 °C per decade; since the mid 1960s that rate has increased to 0.4 °C per decade (ACIA 2005). The North Slope of Alaska has warmed even faster than the rest of the Arctic during the past few decades; Shulski and Wendler (2007) report an increase of more than 3 °C over the past 60 years or 0.5 °C per decade. The coastal town of Barrow, some 310 km northwest of the Toolik site, has warmed significantly (p < 0.01) over the last 60 years with a temperature increase of ~3.1 °C or 0.5 °C per decade (Fig. 2) (Alaska Climate Research Center 2015).

In contrast to the Arctic and North Slope trends, a linear trend analysis of the Toolik datasets revealed no significant trend (p > 0.05) in the ~25-year record of SAT from 1989 to 2010 (Cherry et al. 2014) or in SAT from 1989 to 2014 (Fig. 2). This inability to detect a significant trend (p > 0.05) for these dates also occurred for the Barrow record for the same short period (Fig. 2). The lack of significant warming is also apparent in a closer analysis of the Toolik record for winter, spring, summer, and fall (Fig. 3).

In contrast, the Zackenberg annual air temperatures and the summer temperatures (Figs. 2, 3) show a significant (p < 0.01) warming. Schmidt et al. (2012) report that over the 1997–2008 period, the measured average summer temperature increased dramatically resulting in an increase of between 1.8 and 2.7 °C per decade (p < 0.01), while precipitation data show no significant trends for annual averages or for summer months. To extend the Zackenberg climate database, Hansen et al. (2008) used data from a nearby meteorological station (established in 1958) and from elsewhere in Greenland to create a dataset and calculate a long-term increase in average annual temperature for the period 1901–2005 of 1.39 °C (p < 0.01) and for 1991–2005 of 2.25 °C (p < 0.01); they mention that these trends are similar to trends from other studies along the east coast of Greenland.

### Permafrost temperatures

The variability of SAT from year to year makes it difficult to discern small changes over less than one or two decades. However, as Lachenbruch and Marshall (1986) noted, as the temperature signal moves deeper into the soil the annual variability is filtered out so that temperatures at a depth of 20 m do show a regular trend (Smith et al. 2010). At Galbraith Lake 20 km south of Toolik Lake, permafrost temperatures at 20 m have increased by about 0.8 °C over the past 20 years (Smith et al. 2010, Fig. 4). However, Stieglitz et al. (2003) show that on the North Slope some permafrost warming, perhaps as much as 50%, could be contributed by an increase in snow depth, which insulates the soil from cold winter temperatures.

**Fig. 4:**
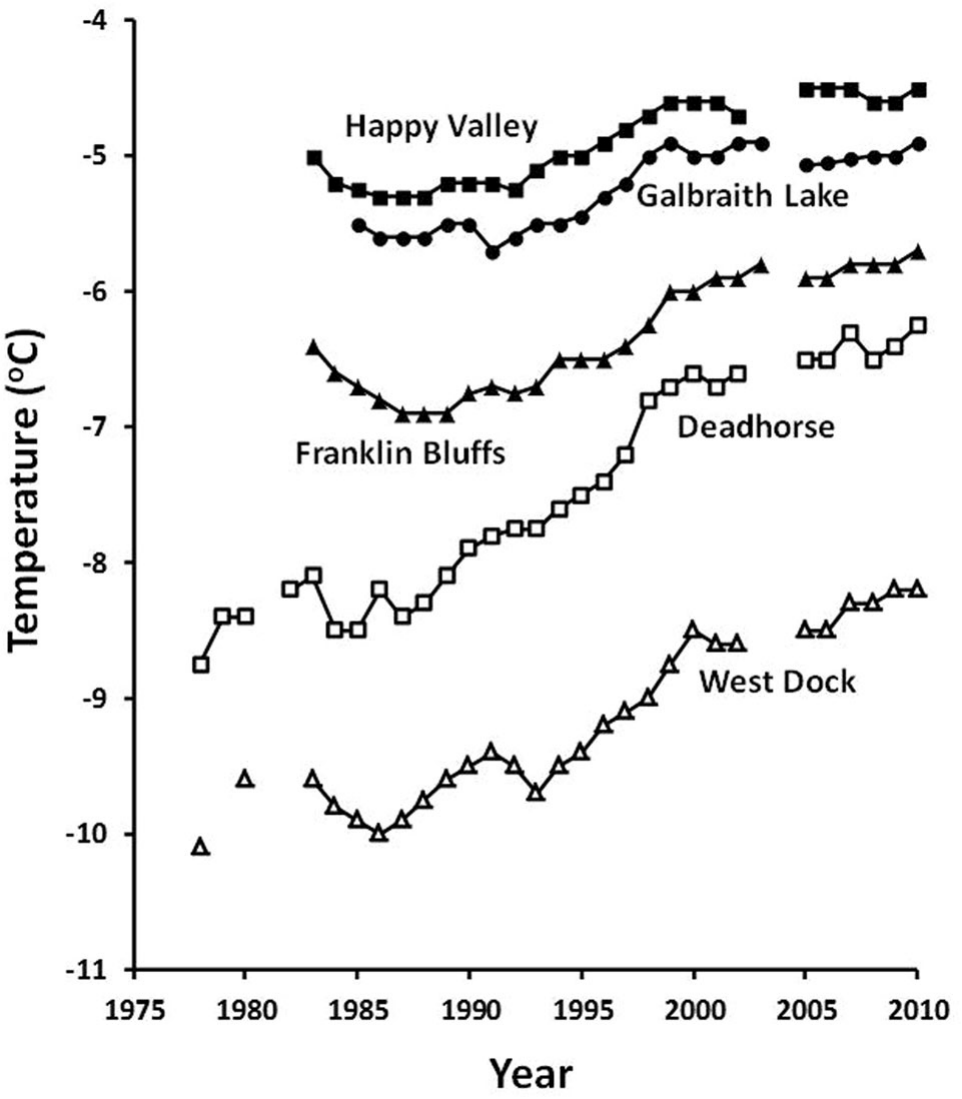
The time series of permafrost temperatures measured by Romanovsky and Osterkamp. Temperatures measured annually at 20 m depths in boreholes along the Dalton Highway south of Prudhoe Bay, Alaska. Locations are the following: West Dock 70^o^18′N, 148^o^25′W; Deadhorse 70^o^11′N, 148^o^27′W; Franklin Bluffs 70^o^00′N, 148^o^40′W; Galbraith Lake 68^o^29′N, 149^o^29′W; Happy Valley 69^o^09′N, 148^o^49′W

From Zackenberg, there are no permafrost temperature data below 1.3 m (Christiansen et al. 2008).

### Changes in depth of active layer thaw

#### Direct measure of depth of thaw with steel probes

**Fig. 5:**
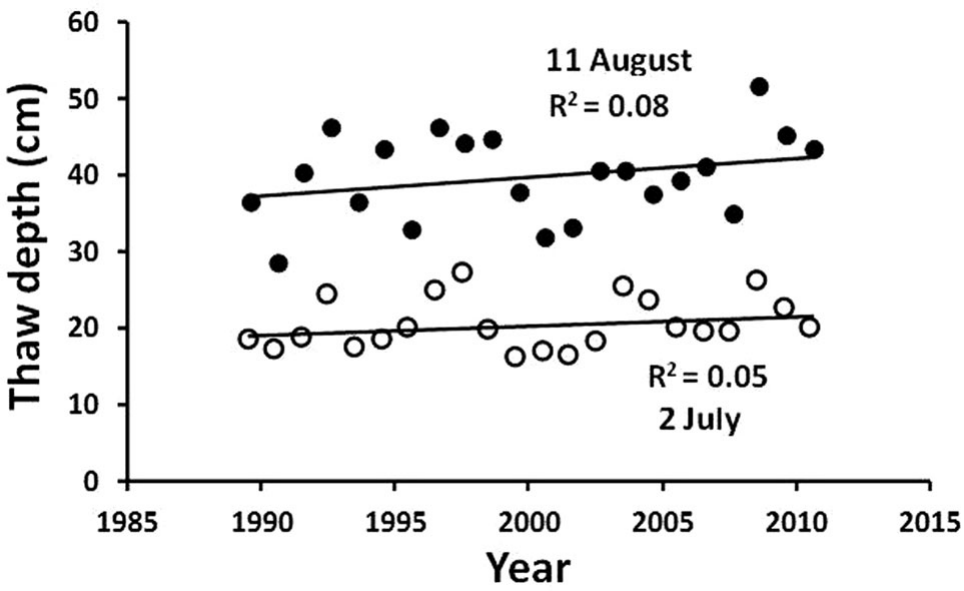
Summer thaw depth (*active layer*) in moist acidic tussock tundra at Toolik Field Station sampled on 11 August (*closed circles*) and 2 July (*open circles*). Figure redrawn from Kling et al. (2014)

The summer depth of thaw of the active layer of the soil is primarily influenced by the surface temperature and the length of the thaw season (Hinzman et al. 2005), snow cover (Stieglitz et al. 2003), the topographic position, soil moisture, thickness of the organic and litter layers, and the structure of the vegetation canopy (Shaver et al. 2014). The mean maximum thickness of the active layer at the Toolik transect in August varies from 28 to 52 cm, and there is no statistically significant trend in thickness or in maximum thaw depth over the 22 years of record (Fig. 5). Shiklomanov et al. (2010) examined a continuous time series of soil thaw measures at Barrow (1994–2009) and also found no apparent trend.

The Zackenberg data, in contrast, show a significant increase (p < 0.01) in the maximum depth of thaw in a 10-year record at ZEROCALM-1 (Christiansen et al. 2008) which varied slightly from 60 to 65 cm in the first 5 years and then increased steadily from 60 to 79 cm over the last 5 years in response to the significant increase in summer temperatures (Fig. 3).

#### Indirect measures of depth of thaw: Chemical measures of soil weathering

**Fig. 6:**
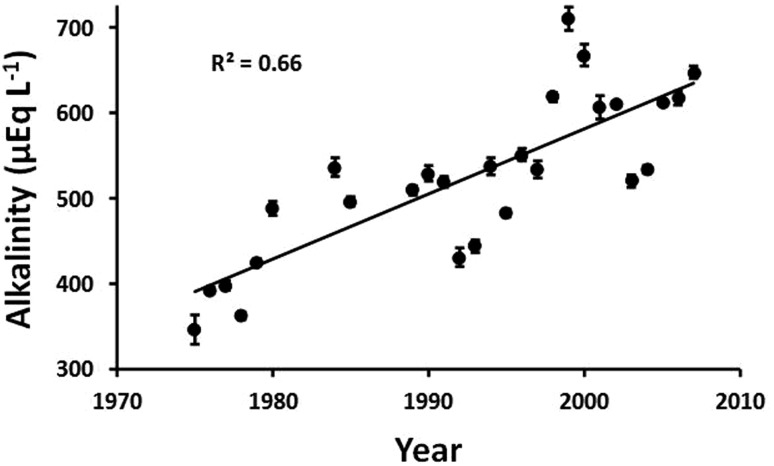
The mean summer alkalinity in Toolik Lake with *error bars* showing the standard errors of the mean. Figure redrawn from Kling et al. (2014)

There is at Toolik, however, additional evidence for an increase in the thickness of the active layer in at least some portion of the catchment. A doubling in the alkalinity has occurred in lake and stream waters (Fig. 6; Hinzman et al. 2005; Kling et al. 2014). This doubling of alkalinity is balanced primarily by changes in dissolved calcium and magnesium (Hobbie et al. 2003). The most likely cause of the doubling is an increase in the weathering of previously frozen mineral soils as water flows at the bottom of the active layer or through new subsurface water-flow pathways. An extreme example of this process did occur in a small stream in the Toolik Lake watershed (Hobbie et al. 1999). This stream passes through a site where several meters of gravel deposited some 10,000 years ago at the edge of a glacier were removed for road construction in the 1970s. The newly exposed surface, previously frozen in permafrost, soon developed an active layer and weathering took place; as a result in 1992–1997, the stream supplied 35% of the phosphate (weathered from Ca_3_(PO_4_)_5_ in the soil) entering Toolik Lake but only 10% of the water.

**Fig. 7:**
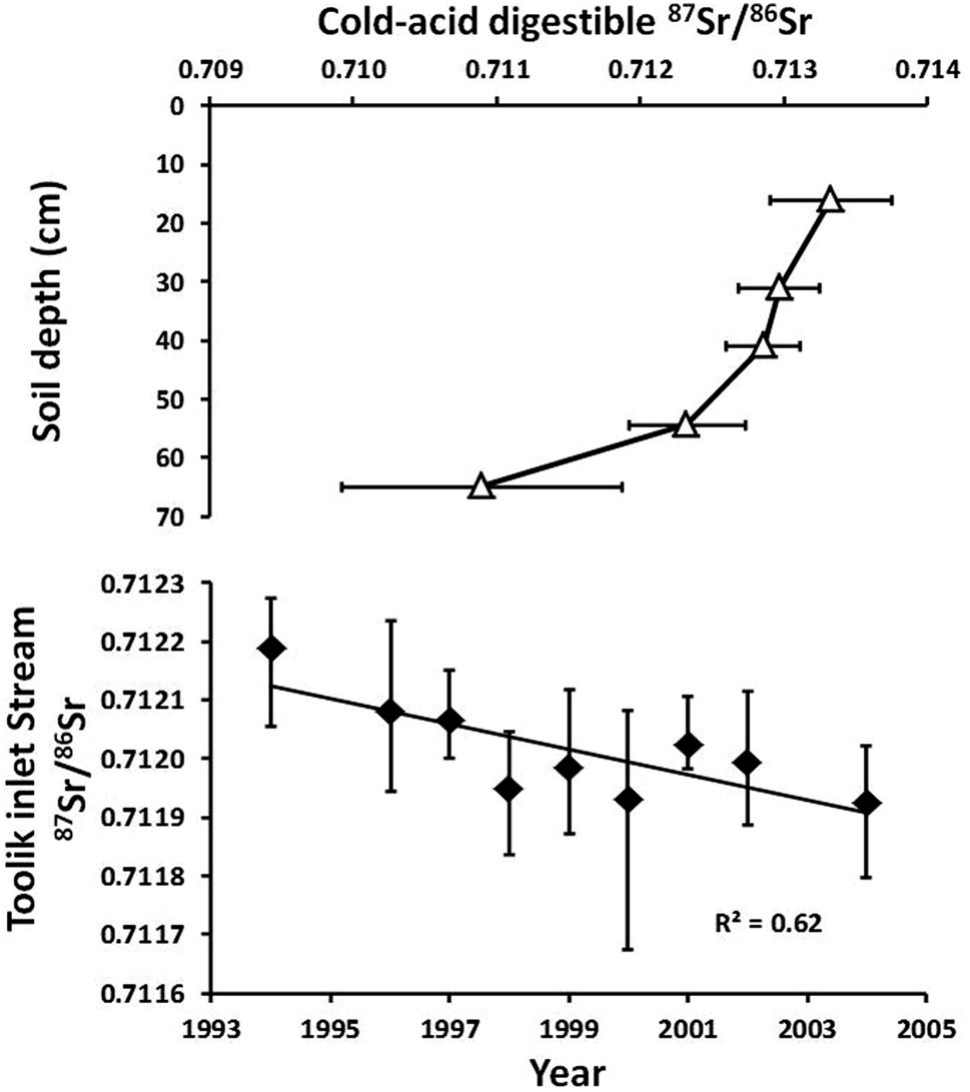
The ratio of strontium isotopes with depth in soils of the most recent glaciation near Toolik Lake (*top*). Strontium isotope ratios in the inlet stream to Toolik Lake over time (*bottom*). Original data from Keller et al. (2007, 2010). Figure modified from Kling et al. (2014)

Additional evidence for an increasing depth of thaw at Toolik comes from geochemical tracers (Kling et al. 2014). In soils, the ratio of strontium isotopes (^87^Sr/^86^Sr) decreases with depth (Fig. 7); thus as the depth of thaw of the soils increases, the rainwater moves through soil layers with progressively lower^87^Sr/^86^Sr ratios. This type of decrease in the isotope ratio was observed in the stream entering Toolik Lake over a ten-year period (Keller et al. 2007, 2010) (Fig. 7). Although the isotope method is sensitive enough to detect very small changes in thaw depth over large areas of the watershed, it is uncertain exactly how much of the thaw occurred uniformly throughout the watershed and how much under new water-flow pathways.

The weathering and water movement in the soil that led to both the increase in alkalinity and the decrease in strontium isotope ratios also integrate the chemical signal over several years. This integration occurs because some of the alkalinity that is produced in one year remains in the soil water at the end of the summer and is not released until the thaw of the active layer the next summer. For example, Everett et al. (1996) measured the Ca^2+^ in soil water for 22 days in August and found an average of 31.4 µEq L^−1^ in overland flow (*n* = 3), 79.8 at 20 cm depth (*n* = 21), and 112 µEq L^−1^ at 40 cm (*n* = 21). Rainfall each fall ensured that the active layer was saturated at the beginning of each winter (Hinzman et al. 1996). The next spring, most of the runoff from the watershed occurred from snowmelt in the spring as surficial runoff when the active layer was still frozen (Woo and Steer 1983). The ions that are a part of the soil water are not released until the thaw depth deepens later in the summer (Cornwell 1992).

At Zackenberg (Christiansen et al. 2008), twenty lakes showed no change in chemical conductivity when monitored twice (1997 and 2003). Two of these lakes also showed no changes when monitored every year from 1997 to 2003. It is not known if weathering of the previously frozen soil would show alkalinity and isotopic changes in the Zackenberg stream and lake watersheds in the same way as soils at Toolik.

### Relative species abundance and composition of tundra vegetation

**Fig. 8:**
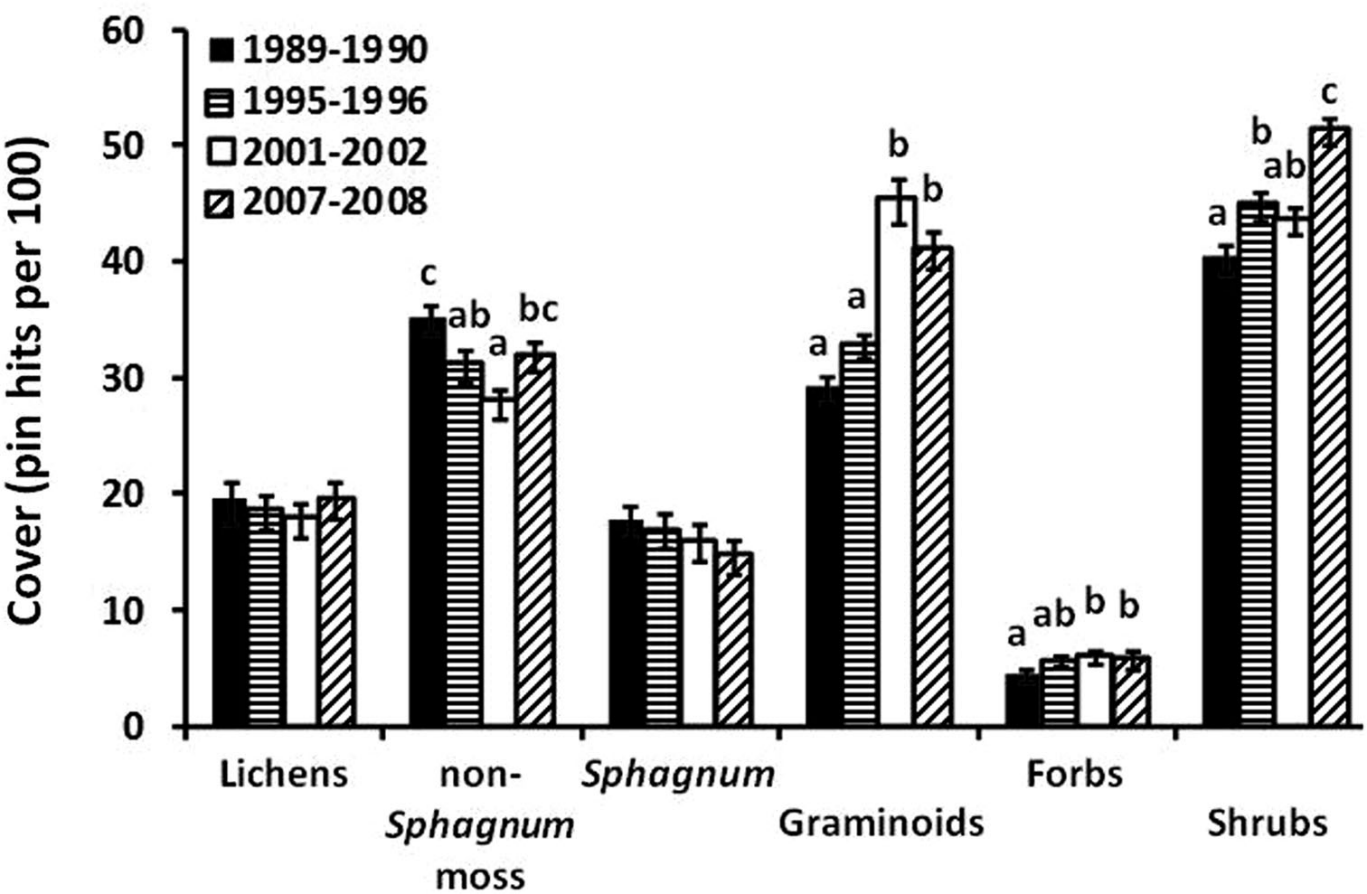
The number of hits per plot of different vegetation growth forms at the Imnavait Creek and nearby Toolik grids. There were a total of 156 plots each sampled four times from 1989 to 2008. The *letters above the bars* indicate significant differences while *error bars* represent standard errors. Statistical differences determined via MANOVA with Tukey’s B post hoc test to determine significant differences among years (p < 0.01). Figure redrawn from Shaver et al. (2014). Original data from Mercado-Díaz (2011)

A number of observers (Sturm et al. 2001; Hinzman et al. 2005; Myers-Smith et al. 2011; Elmendorf et al. 2012) have noted that shrubs in tundra in northern Alaska and in the Arctic as a whole are becoming more abundant. This change is attributed to climate warming as there are no other changes, such as nitrogen deposition, that have occurred in recent times. Toolik point-frame measurements (ITEX) were used for the two decades of measurement (Gould and Mercado-Díaz in Shaver et al. 2014). Over this period, the relative abundance of vascular vegetation increased by 19% (Fig. 8), graminoids increased by 25.5%, herbaceous dicots by 24%, and shrubs by 13%: all increases were significant (p < 0.05). Both canopy height and the horizontal extent of an upper canopy, which overshadows ground layer vegetation, increased. An increase in multiple canopy layers from 60 to 80% represents greater structural complexity of the vegetation and is primarily due to growth in the shrub *Betula nana* and the graminoids *Eriophorum vaginatum* and *Carex bigelowii*. In contrast, the relative abundance of the nonvascular vegetation decreased significantly (p < 0.05): lichens by 9.3%, non-*Sphagnum* mosses by 20%, and *Sphagnum* by 28%. This positive response of plant growth to warming is similar to that found throughout the Low Arctic (Elmendorf et al. 2012).

The ITEX protocol was also used twice at Zackenberg to measure changes in the eight dominant plant communities from 1997 to 2008 (Schmidt et al. 2012). Each community had four replicate sampling plots. In contrast to the Toolik results, there were significant reductions of up to 55% in the cover of grasses and lichens across all plant communities. Yet, some species and groups, including the willow (*Salix arctica*), exhibited only minor changes during this period. The interpretations suggested for Zackenberg by Schmidt et al. (2012) for point-frame analysis and Campioli et al. (2013) for heating experiments are that some of the reductions may be due to the lower sensitivity of High Arctic plant communities to warming than those in the Low Arctic or High Arctic communities could even be resistant to climate change. However, a complicating factor was reduced availability of water during the summers caused by deepening of the active layer. In addition, there was little sign of the marked expansion of shrubs found in most of the Low Arctic (Walker et al. 2006) but musk oxen grazing (Forchhammer et al. 2005) and the relatively short period of observations might make it difficult to measure any expansion.

### NDVI measures of plant biomass

#### NDVI for the Toolik region in northern Alaska

**Fig. 9:**
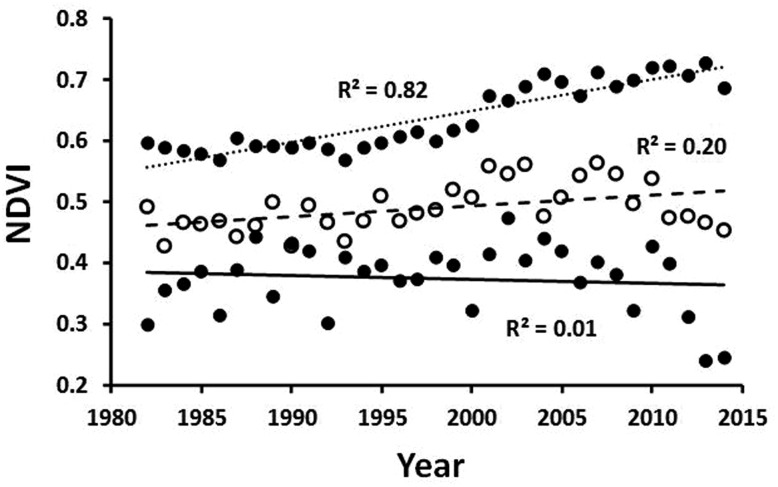
Peak NDVI for a ~1260 km^2^ area centered on the Toolik Field Station site. The dates for the spring (*solid lines*, *closed circles*), summer (*dotted lines*, *closed circles*), and *fall dashed line*, *open circles*) are May 1–June 15, June 16–August 15, and August 16–September 30, respectively. Data provided by K. Guay

The NDVI of the Toolik region (Fig. 9) measures a region of tussock-sedge, dwarf-shrub, and moss tundra on the Circum-Arctic Vegetation Map (Walker et al. 2005). Between 1982 and 2014, the peak season NDVI (±1 SD) significantly increased by 29%, from 0.56 to 0.72 (±0.055) (p < 0.001). For the same period as the point-frame measures of biomass (1989–2008), the NDVI increased by 17%, which is close to the point-frame values of a 19% increase in total vascular vegetation and a 13% increase in shrub abundance (Fig. 8). The fastest increase in NDVI is in the summer but there is also a significant increase in the fall period, from 0.46 to 0.52 (±0.055) (p < 0.01). Spring NDVI actually declined somewhat over the 33-year period although the trend was not statistically significant. Previous satellite assessments (e.g., Jia et al. 2003; Verbyla 2008; Beck and Goetz 2011), which covered larger areas of northern Alaska but over shorter time periods, also showed a systematic increase in the NDVI in recent decades. Bieniek et al. (2015) report NDVI data for the Alaskan coastal zone west of Barrow that are very similar (1982–2013) to the Toolik values (Fig. 9); they also measured a decrease in the spring.

The NDVI for the Toolik region has also been analyzed at much finer scales by Raynolds et al. (2013) who used six scenes from Landsat 4 or more-recent sensors (1985–2007) showing the annual peak NDVI as measured at a 30-m pixel resolution over an 823 km^2^ area. They analyzed changes in 14 types of vegetation and found that nearly all the patches showed either no increase or a small increase in NDVI; in fact, sizeable increases in NDVI were found only in tussock tundra, non-tussock-sedge tundra, and acidic dwarf-shrub tundra, the latter making up only 5% of the pixels. Thus, the increase in NDVI evident at a coarser scale (Fig. 9) was also present at the finer scale but was heterogeneously distributed. Further comparisons between the AVHRR (Fig. 9) and the Landsat values (Raynolds et al. 2013) are difficult because NDVI values measured with different sensors and at different levels of resolution and types of rectification may be quite different (Goetz 1997).

The changes in NDVI (Fig. 9) indicate a regional increase in vegetation photosynthetic activity and aboveground plant biomass. The plot measurements of plant and leaf biomass at the Toolik site (Fig. 8) indicate that this biomass increase is largely the result of increased growth by deciduous shrubs (e.g., dwarf birch, willows, and alder) in response to multi-year warming, but this response is shared with graminoids and forbs. Several researchers attribute the slow increase in biomass to a slow increase in the availability of N to plants (Shaver et al. 1992, 2014; Pearce et al. 2015; Jiang et al. 2015). It is well known through warming and fertilization experiments that the N supply strongly limits plant growth in northern Alaska and that warming increases the microbial mineralization of organic nitrogen in the soil, the major source of N to plants in the tundra.

#### NDVI for the Zackenberg region in Greenland

At Zackenberg (Tagesson et al. 2012), the annual maximum NDVI increased from 0.35 to 0.61 between 1992 and 2004, an increase of 74%, before dipping to 0.49 in 2005 and returning to 0.57 in 2007 and 2008. The authors suggest that this dip in the NDVI could have been caused by a one-year change in the satellite sensor. In any case, the increase in NDVI at Zackenberg is consistent with other studies of NDVI trends in the Arctic, including the Toolik data (Fig. 9), that interpret the trend as an increase in both greening and plant photosynthesis (see also Jia et al. 2003; Verbyla 2008). However, the rates of change of the Zackenberg NDVI are much higher (0.02/year) than rates in other studies (0.003–0.006/year). Tagesson et al. (2012) suggested the high rates may be due to the almost complete plant cover in the 1.4 km^2^ study area as compared with other arctic study areas that typically include a large fraction of gravel, rock, and water.

As with the permafrost temperatures at 20 m, vegetation responses to temperature are integrated over several years and thereby damp out the hourly, daily, and seasonal variation in the noisy air temperature signal. Plants accumulate and retain biomass and nutrients from year-to-year and decade-to-decade. Thus, the effects of an unusually cold or warm summer are damped out but the long-term trend in temperature is preserved in long-lived tissues such as rhizomes, woody stems, and branches. Results of small but persistent changes, like the warming signal, accumulate over decades and become detectable because these long-lived tissues integrate effects over many years and are not reset to a base level each year.

### Ecosystem and biota temporal trends, Zackenberg

As noted in the description of Zackenberg ecosystem research, one type of long-term study is the detailed analysis of changes across the entire aquatic and terrestrial food webs. In contrast, at the Toolik site, the long-term studies are only of selected parts of the entire ecosystem, for example, the point-frame measures of changes in the plant community types described above.

From 1996 to 2010, studies were made of trends and temporal variations across the whole aquatic and terrestrial ecosystem. In the terrestrial ecosystem, 6 plant species, 6 taxa of arthropods, 4 species of birds, and 3 species of mammals were measured (Mortensen et al. 2014). Plants were sampled weekly for such factors as abundance, flowering dates, and emergence, arthropods were sampled in traps, and mammals by census. Of the biotic variables, 39% exhibited significant linear trends; of the significant trends in the abundance of terrestrial biota, 12 had a positive slope and 22 had a negative slope. The Zackenberg data also revealed (Høye et al. 2013) that warmer summer temperatures led to a shortening of the season of plant flowering and likely to a decline in the abundance of the insects that visit these plant flowers for basking, nectar and pollen feeding, mating, and ovipositioning. For the aquatic ecosystems, both the species and abundance of phytoplankton and zooplankton responded strongly to water temperatures that varied from 4 to 11 °C from one summer to the next. One lake was dominated by chrysophytes in a warm summer (95% of abundance) and by dinophytes (96% of abundance) in a cold summer.

### Filtering long-term data to determine environmental trends

#### Low Arctic Toolik

A large number of multi-year measurements are being collected at the Toolik site (e.g., Table 2) but only permafrost temperatures, plant biomass, and chemical measures of soil weathering (strontium ratios and lake alkalinity) revealed changes attributable to climate warming (Table 3). Why were these three measures useful indicators of change while others were not? One way to think about this problem is in terms of a filtration effect. In these useful measures, there is something that occurs during the generation of the signal or in the analysis that filters or reduces the noise around the signal so that trends can be detected over a reasonable period of time. For the Toolik dataset, which is only 25 years long, the best type of filter is a “medium-pass” filter that accumulates the effects of the changing climate over several years to reveal trends due to decadal changes. In contrast, a “high-pass” filter responds strongly to variation in climate on short-time scales (<few years) and a “low-pass” filter responds so slowly to climate change that the change is undetectable on a decadal time scale.

**Table 3:** Toolik and Zackenberg long-term monitoring results

Variable	Characteristics	Toolik	Zackenberg
Air temperature	Useful for long-term series (decades) of moderate warming Useful for shorter-term series(<decades) in case of intense warming	No significant trend because of short record and high annual variability	Significant trend related to large annual warming
Active layer thickness	Is reset every year so closely related to summer temperature	No long-term significant trend in thickness	Significantly thicker
	Changes in surface water alkalinity are evidence of deeper penetration of water into soil previously permanently frozen	Doubling of alkalinity in lakes is evidence that water is penetrating deeper into soil as active layer increases	No change in surface water chemistry
Permafrost temperature at 20 m	As temperature signal moves deeper into the soil, the high-frequency noise is filtered out	Increase of 0.8 °C over long-term	No measures made at depths >1.2 m
Plant and animal abundance with point-frame and species counts	Abundance of individual terrestrial and aquatic species or of communities	Shrubs, graminoids increase	Some species increase, most decrease but grasses and lichens decrease
NDVI satellite measure of peak greenness at annual or seasonal periods	Estimate of peak annual or seasonal biomass or photosynthesis. Integrates over various areas	Steady increase 1982–2014	Increase 1992–2004, instrument changes to 2008

**Fig. 10:**
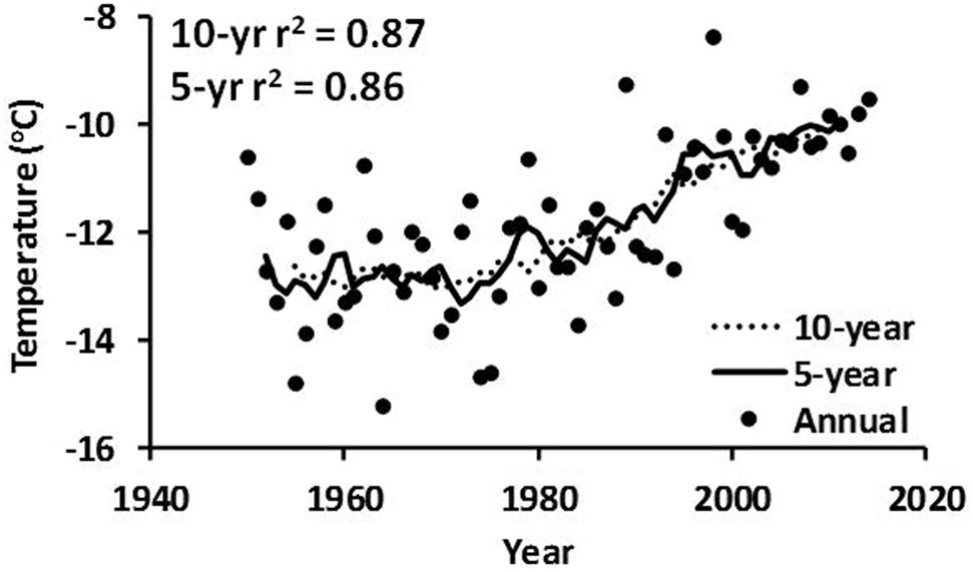
The annual mean air temperature and the 5- and 10-year running mean values for the Barrow data shown in Fig. 2. The *R* ^2^ for the 5-year running mean is 0.86 and for the 10-year running mean is 0.87. Data sources as in Fig. 2

An example of the use of a filter is the moving average (Chatfield 2004) commonly used for long-term air temperature analysis. This method was used with a 10-, 20-, and 30-year filter to analyze the mean summer air temperatures for the Italian Central Alps (1610–2008; Coppola et al. 2013). For northern Alaska, a 5- and 10-year moving average produced smoothed curves with significant *R* ^2^ values for the Barrow data (Fig. 10). Unfortunately, the Toolik data cover only 25 years and have missing data points and a high variability; therefore, the air temperature trend is not significant (p > 0.01) (Fig. 2) and the moving average cannot be used.

The permafrost record of temperature at 20 m depth in the soil (Fig. 4) is an excellent example of naturally occurring moving average or running mean, and no manipulation of the data is needed. As Lachenbruch and Marshall (1986) point out, as the temperature signal moves deeper into the soil, the high-frequency noise is progressively filtered out. However, as previously noted, some of the change in snow depth will also affect soil temperatures so that permafrost temperature change reflects the temperature of the upper layers of the soil and not just the air temperature during climate change (Stieglitz et al. 2003).

Another way that the natural environmental measures act as filters to reduce the variability from year to year is by accumulation of the effects. That is, some of the environmental effects of one year are stored and affect the data for the next year or years. The plants that are most useful for tracing warming (Figs. 8, 9) have large amounts of biomass in their stems, branches, and roots; these tissues persist from year to year such that small warming-induced increments in biomass accumulate over time. The plants also translocate and store nutrients and carbohydrates over winter for use in leaf production the next spring, so that resources for growth that are acquired in one-year affect subsequent year’s growth. In much the same way, the alkalinity that is added to soil water as a product of weathering is not completely flushed out at the end of the summer and may persist in the soil for years before it is transported to lakes (Fig. 6).

A part of understanding the concept of environmental and ecological filters is the notion that some measurements are not useful as indicators of change because they are reset to the same level every year. For example, the depth of thaw of the soil at Toolik is measured at its summer peak (Table 2; Fig. 5) but is reset to zero centimeters each winter when the entire active layer is frozen. Similarly, summer temperatures in lakes, chlorophyll content of lake plankton, or temperature of the Kuparuk River are all reset at the start of each year.

#### High Arctic Zackenberg

These insights about environmental filters developed from the Low Arctic site at Toolik do not really agree with the results of long-term environmental studies at the High Arctic site at Zackenberg. Out of all the ecological changes that occurred at this High Arctic site only one, the change in canopy reflectance or NDVI, appeared to be the result of integration of a climate change effect from one year to another. This integration, which occurs because plants retain biomass, nutrients, and carbohydrates from one year to another, decreases signal noise and acts as a medium pass filter.

A possible reason for the difference between these sites is the consistent and high rate of warming occurring during the period of research at Zackenberg when the summer average temperatures increased between 1.8 and 2.7 °C/decade (p < 0.01) (Schmidt et al. 2012). At Toolik, there was no significant trend of summer and of annual average temperatures and at least summer temperatures were more variable (Fig. 3). However, at Zackenberg, the dramatic rise in the summer temperatures has increased the thickness of the active layer, increased the overall plant biomass or productivity as measured by NDVI, and increased numbers or biomass of some of the biota while decreasing the numbers or biomass of others. For example, at Zackenberg, there was a large reduction of the biomass of grasses and lichens but no increase in shrubs. Evidently some plants and plant communities are sensitive to change while others have a high degree of resistance.

## Conclusions

An overall warming trend does occur in northern Alaska where air temperatures at Barrow, the only permanent town, have warmed ~3.1 °C in the last 65 years. However, at the Toolik site, the 25-year record of annual mean air temperatures revealed no significant trend due to the high variability from year to year and the relatively short period of record. In spite of the temperature variability and the absence of a warming trend, three of the Toolik measurements did produce evidence of change: a warming of permafrost temperatures at a depth of 20 m, an increase of plant biomass as measured on the ground and by means of satellite NDVI, and a change in surface water chemistry indicating an increase in weathering of previously frozen soil. These indicators have the common feature of integrating effects of the climate signal on multi-year to decadal time scales. They act as a medium pass filter that reduces the signal variability yet allows the effects of long-term warming to emerge within the 20- to 30-year dataset. Based on the indicators that have passed through the medium pass filter, we conclude that there has been a measureable response of the Toolik ecosystem to arctic warming, even though there has been no statistically significant warming trend in the annual air temperature.

The concept of the medium pass filter, developed from the Low Arctic Toolik dataset, has only limited application for analyzing results from the High Arctic at Zackenberg in northern Greenland. At this site, the summer temperatures are increasing at an unusually high rate. Some results of the environmental measurements, such as the thickness of the active layer and abundance of some plants, increase in parallel with the warming temperatures. Others, such as biomass of grasses and lichens, have dramatically decreased. The NDVI measure does increase in a similar manner in the High Arctic and Low Arctic sites, and it is likely that there is a medium pass filter at work in which some integration is achieved because of year-to-year carryover and reuse of plant biomass, nutrients, and carbohydrates. It would be interesting to see results of temperature measures at 20 m depth where the soil acts to filter out the year-to-year variability.

## Footnotes

1
http://arc-lter.ecosystems.mbl.edu.

2
http://data.g-e-m.dk.
